# Supplementary material for: Lensfree OLEDs with over 50% external quantum efficiency via external scattering and horizontally oriented emitters
Source: Nat Commun. 2018 Aug 10;9:3207. doi: 10.1038/s41467-018-05671-x (PMC6086904; doi:10.1038/s41467-018-05671-x)
Supplement: Supplementary file 1 — Supplementary information [file 41467_2018_5671_MOESM1_ESM.pdf]

Supplementary Information

Lensfree OLEDs with over 50% external quantum efficiency via external scattering and horizontally oriented emitters

Jinouk Song[1], Kwon-Hyeon Kim[2], Eunhye Kim[1], Chang-Ki Moon[2], Yun-Hi Kim[3], Jang-Joo Kim[2]*, and Seunghyup Yoo[1]*

[1] *School of Electrical Engineering, Korea Advanced Institute of Science and technology (KAIST), Daejeon 34141, Republic of Korea.*

[2] *Department of Materials Science and Engineering, Seoul National University, Seoul 151-744, Republic of Korea.*

[3] *Department of Chemistry and Engineering Research Institute (ERI), Gyeongsang National University, Jinju 66-701, Republic of Korea.*

*To whom all correspondence should be addressed: E-mail: syoo@ee.kaist.ac.kr, jjkim@snu.ac.kr

a

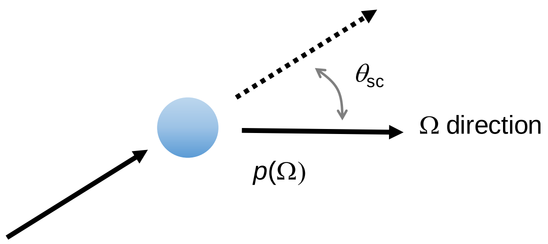

b

$T_{inline} = \exp(-\mu_{sc} d_{SL})$ (absorption-free)

$\mu_{sc} = 1/L_{MFP}$

$S = d_{SL}/L_{MFP} = \ln(1/T_{inline})$

where μ_{sc} is scattering coefficient, d_{SL} is the thickness of a scattering layer, and L_{MFP} is mean-free path.

Supplementary Figure 1 | Definition of asymmetry parameter (*g*) and scatterance (*S*)[1]. (a) Schematic diagram illustrating the meaning of asymmetry parameter (g), which is defined in average as the cosine of the scattering angle (θ_{sc}) weighted by phase function $p(\Omega)$: $g = \langle \cos\theta_{sc} \rangle = \int_{4\pi} p(\Omega)\cos\theta_{sc} d\Omega$ where Ω is solid angle. **(b)** Summary of the equations relating key scattering parameter.

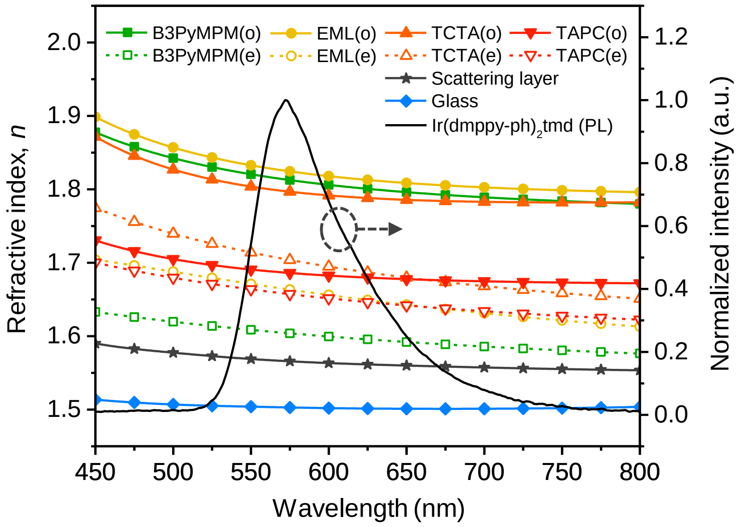

Supplementary Figure 2 | The optical constants of materials used in optical simulation. Refractive indices of organic materials, a glass substrate, and the host material of a scattering layer (NOA 73) as well as the emission spectrum of Ir(dmppy-ph)$_2$tmd used in global optimization are shown. Ordinary and extraordinary refractive indices ((o) and (e), respectively) of all organic materials are measured by variable angle spectroscopic ellipsometry (VASE) using alpha-SE Ellipsometer (J.A. Woollam Co.). Photoluminescence (PL) spectrum of Ir(dmppy-ph)$_2$tmd is also shown as a solid line[2].

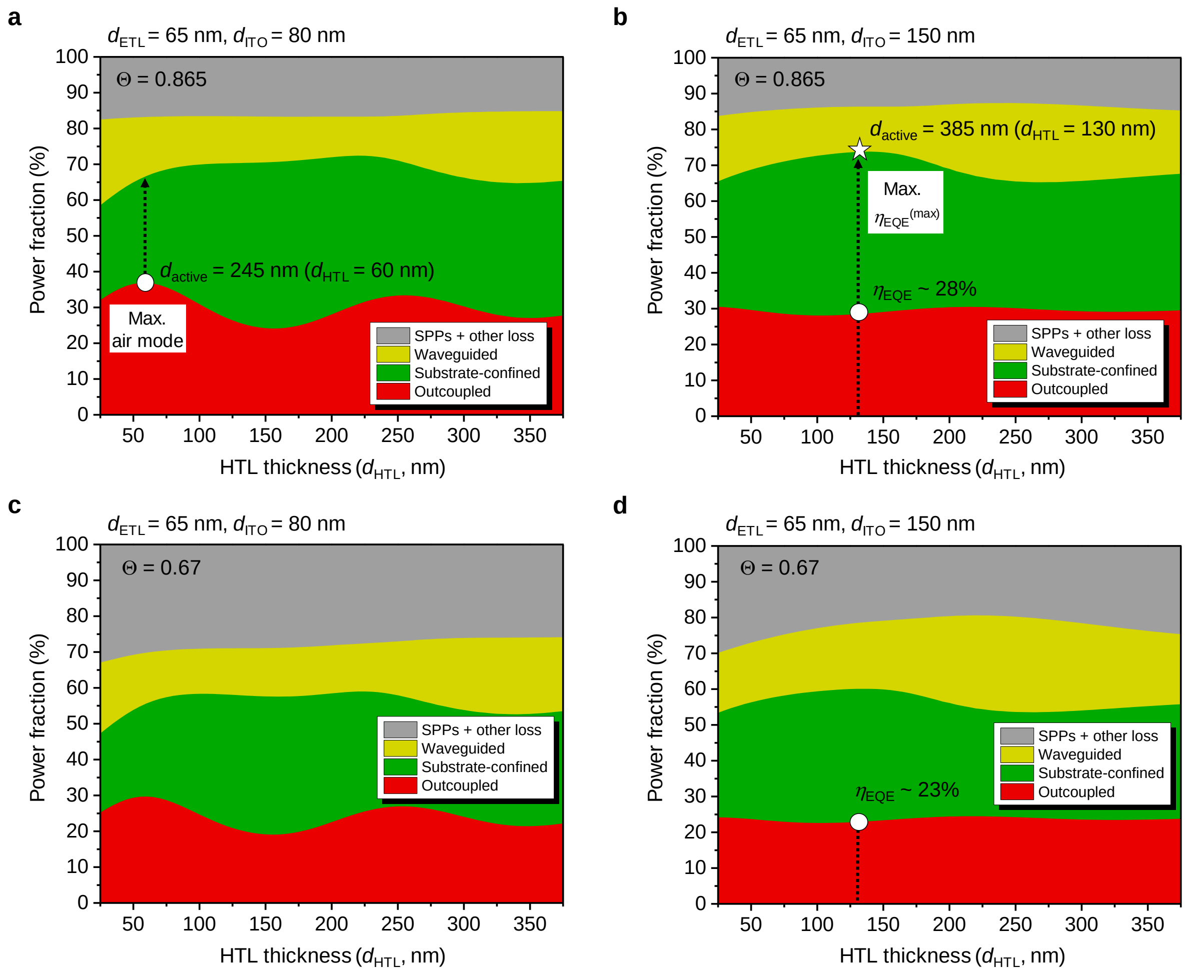

Supplementary Figure 3 | The effect of total active layer thickness (d_{active} = d_{ITO} + $d_{org.}$) on the power fraction. (a-b) Power dissipation spectra of the reference device obtained for **(a)** d_{ITO} = 80 nm and **(b)** d_{ITO} = 150 nm. Here, d_x denotes the thickness of layer 'x' while 'org.', ITO, ETL, and HTL refer to organic, indium tin oxide, electron and hole transport layers, respectively. The case shown in (a) contains the global maximum air mode condition (d_{active} = 245 nm with d_{ITO} = 80 nm) with d_{ETL} = 65 nm without a scattering layer, and the case shown in (b) contains the condition maximizing the sum of air and substrate modes (d_{active} = 385 nm with d_{ITO} = 150 nm). The latter is found to correspond to the condition that eventually leads to the global maximum when the proposed scattering layer is used together. All the results in (a) and (b) are for OLEDs based on Ir(dmppy-ph)$_2$tmd, which has horizontal dipole ratio (Θ) of 0.865. **(c-d)** Power dissipation spectra of the reference device for **(c)** d_{ITO} = 80 nm and **(d)** d_{ITO} = 150 nm are presented also for an emitter same as Ir(dmppy-ph)$_2$tmd but having Θ of 0.67 (i.e. random, isotropic dipole orientation).

a

$$Q_{sc} = \frac{\text{scattering cross section } (\sigma_{sc})}{\text{geometrical cross section}} = \frac{\sigma_{sc}}{(\pi d_{SP}^2/4)}$$

$$\mu_{sc}^{(c,max)} = N_{SP}^{(c,max)}\sigma_{sc} = \sigma_{sc} / d_{SP}^3$$

$$= Q_{sc}\,(\pi d_{SP}^2/4)/\, d_{SP}^3 = (\pi/4) Q_{sc} / d_{SP}$$

$$= 1/L_{MFP}^{(c,min)}$$

b

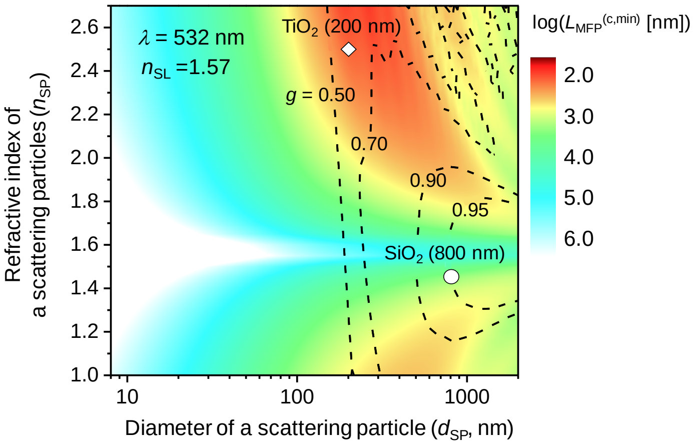

Supplementary Figure 4 | Definition of scattering efficiency and its relation to key scattering parameters (a) The definition of scattering efficiency (Q_{sc}) and its relation to (i) the maximum scattering coefficient (μ_{sc}) achievable for simple cubic arrangement of nanoparticles ($\mu_{sc}^{(c,max)}$) and (ii) the corresponding minimum mean-free path (MFP) [= L_{MFP}] for the same particle arrangement ($L_{MFP}^{(c,min)}$). **(b)** Logarithm value of $L_{MFP}^{(c,min)}$ as a function of the diameter (d_{SP}) and refractive index (n_{SP}) of a scattering particle in the host (NOA 73, n_{SL} ~ 1.57). Note that maximum μ_{sc} and corresponding minimum L_{MFP} can differ from $\mu_{sc}^{(c,max)}$ and $L_{MFP}^{(c,min)}$ because packing density varies depending on the particle arrangement (ex. simple cubic, face-centered cubic, hexagonal close packed, etc.) but should be in the same order of magnitude. Actual μ_{sc} and corresponding L_{MFP} may differ significantly from $\mu_{sc}^{(c,max)}$ and $L_{MFP}^{(c,min)}$ because such closely packed geometries are not easily formed due to processing issues, etc.; nevertheless, these values serve as (i) good guidelines for the upper limit of μ_{sc} and the lower limit of L_{MFP} for a given host / scatterer system or as (ii) a barometer to tell how easily one can achieve a target scatterance.

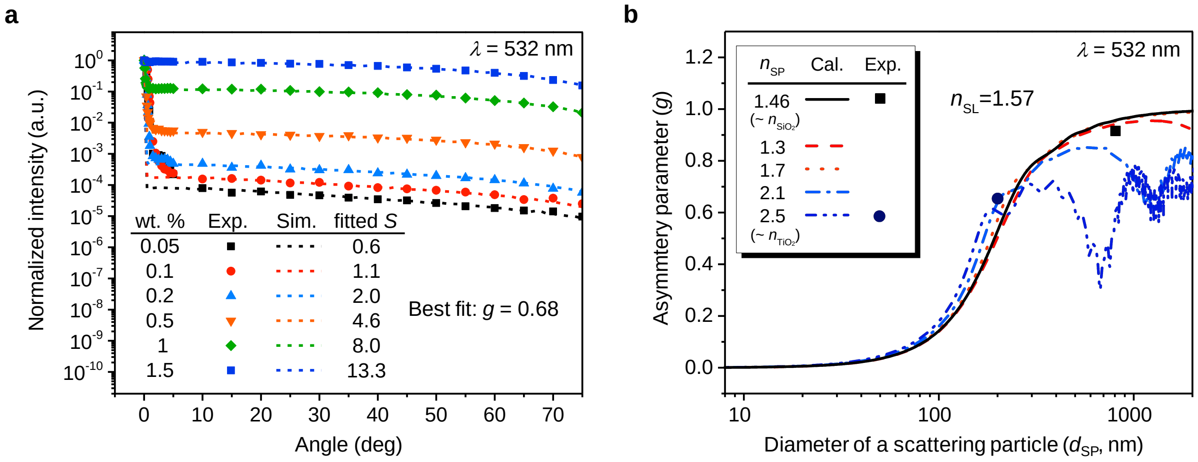

Supplementary Figure 5 | Scattering layers prepared by dispersing TiO_2 nanoparticles (diameter = 200 nm) in NOA 73 host. (a) Measured (dot) and fitted (dashed) graphs of angular characteristic of scattered light after passing through the TiO_2-embedded scattering layers with different level of concentration in wt.%. From the best fit, asymmetry parameter (g) is found to be 0.68. **(b)** The comparison between calculated and obtained asymmetry parameters at the wavelength of 532 nm. Experimental data are for SiO_2 (n_{SP} = 1.46 ; d_{SP} = 800nm) and TiO_2 (n_{SP} = 2.50 ; d_{SP} = 200nm) embedded in the NOA 73 host (n_{SL} = 1.57).

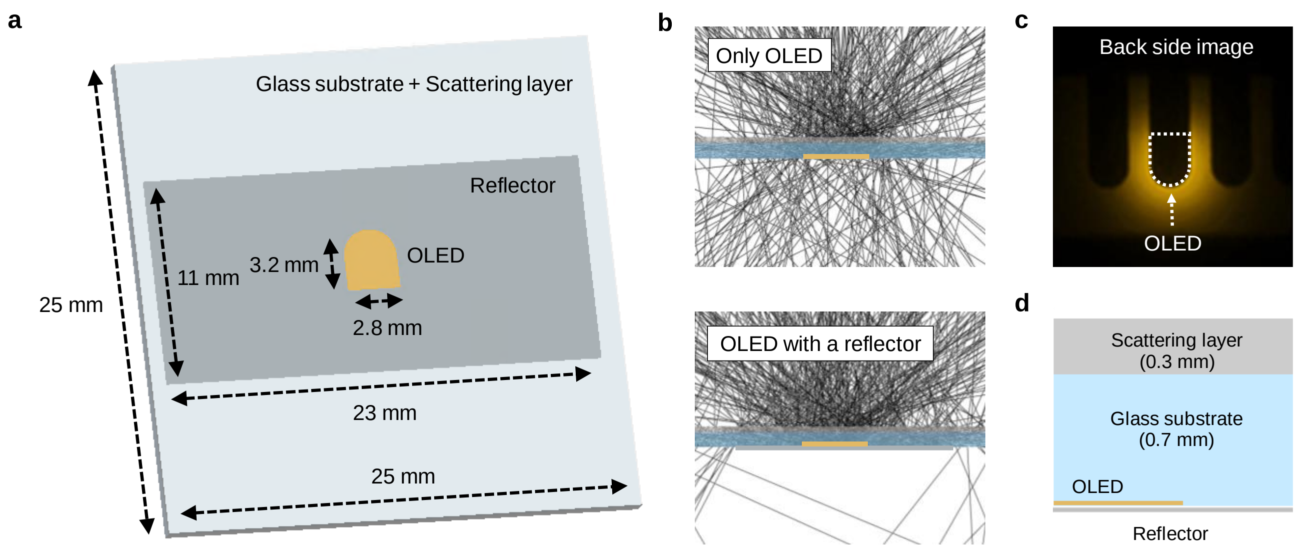

Supplementary Figure 6 | Configurations of a device under study used in LightTools^{TM} simulation. (a) Oblique top view of the geometry (type A in the main text) used in the LightTools^{TM} simulation as well as in the experiment. The orange area is the device active region where emission occurs, and a surrounding gray area illustrates the aluminum reflector, which we placed below the cathode of an organic light-emitting diode (OLED) to reduce the finite size effect, as described in Method Section. **(b)** Side view of simulation ray diagrams comparing without (top) and with the reflector (bottom). The simulation results clearly show that a significant portion of the emitted rays proceeds backward by back scattering. Without the reflector, those rays would pass through the metal-free portion, exiting the device without returning to the scattering layer. With a reflector, such loss is suppressed to a significant degree although a small portion can exit the device in the rear side, unless the reflector. The dimensions in (a) and (b) are for the configuration defined as Type A in Fig. 4 of the main text. **(c)** A photograph taken from the rear side of an OLED with a scattering layer. This photo clearly illustrates that nontrivial amount of light propagates into backward. **(d)** Side view of the simulation geometry.

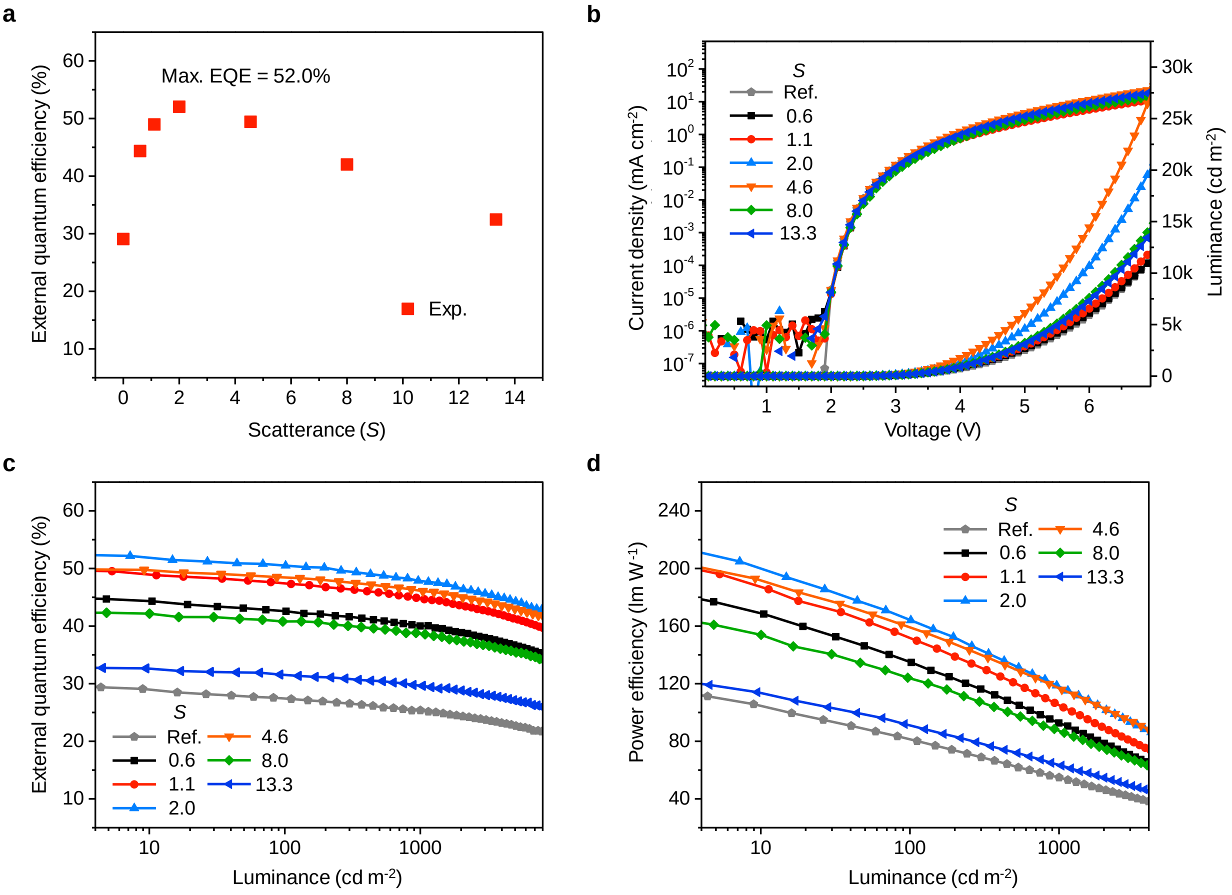

Supplementary Figure 7 | Characteristics of Ir(dmppy-ph)$_2$tmd-based organic light-emitting diodes with TiO_2 scattering layers. **(a)** External quantum efficiency (EQE) as a function of scatterance (*S*) **(b)** Current density (*J*) - luminance (*L*) - voltage (*V*) characteristics. **(c-d)** **(c)** EQE and **(d)** Power efficiency versus *L*. Results shown here are for **Type A** devices having optimized structure: ITO (150 nm) / TAPC (130 nm) / TCTA (10 nm) / TCTA:B3PYMPM:Ir(dmppy-ph)$_2$tmd (4 wt.%, 30 nm) / B3PYMPM (65 nm) / LiF (1 nm) / Al (100 nm).

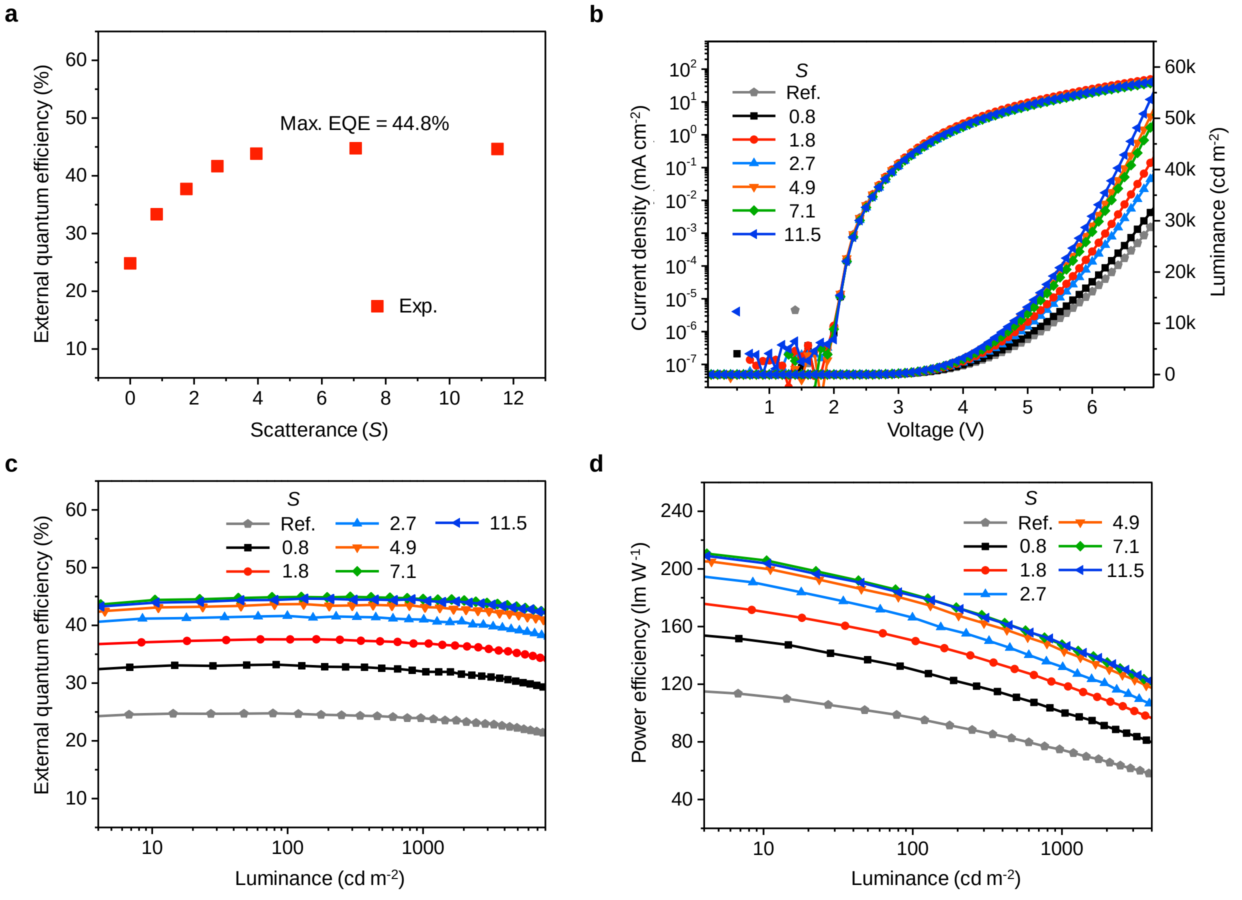

Supplementary Figure 8 | Characteristics of Ir(ppy)$_2$acac-based organic light-emitting diodes with SiO$_2$ scattering layers. (a) External quantum efficiency (EQE) as a function of scatterance (*S*) **(b)** Current density (*J*) - luminance (*L*) - voltage (*V*) characteristics. **(c-d) (c)** EQE and **(d)** Power efficiency versus *L*. Results shown here are for Type A devices having optimized structure: ITO (150 nm) / TAPC (90 nm) / TCTA (10 nm) / TCTA:B3PYMPM:Ir(ppy)$_2$acac (8 wt.%, 30 nm) / B3PYMPM (65 nm) / LiF (1 nm) / Al (100 nm). Note that Ir(ppy)$_2$acac has a slight preference toward horizontal dipole orientation (Θ) being 0.76.

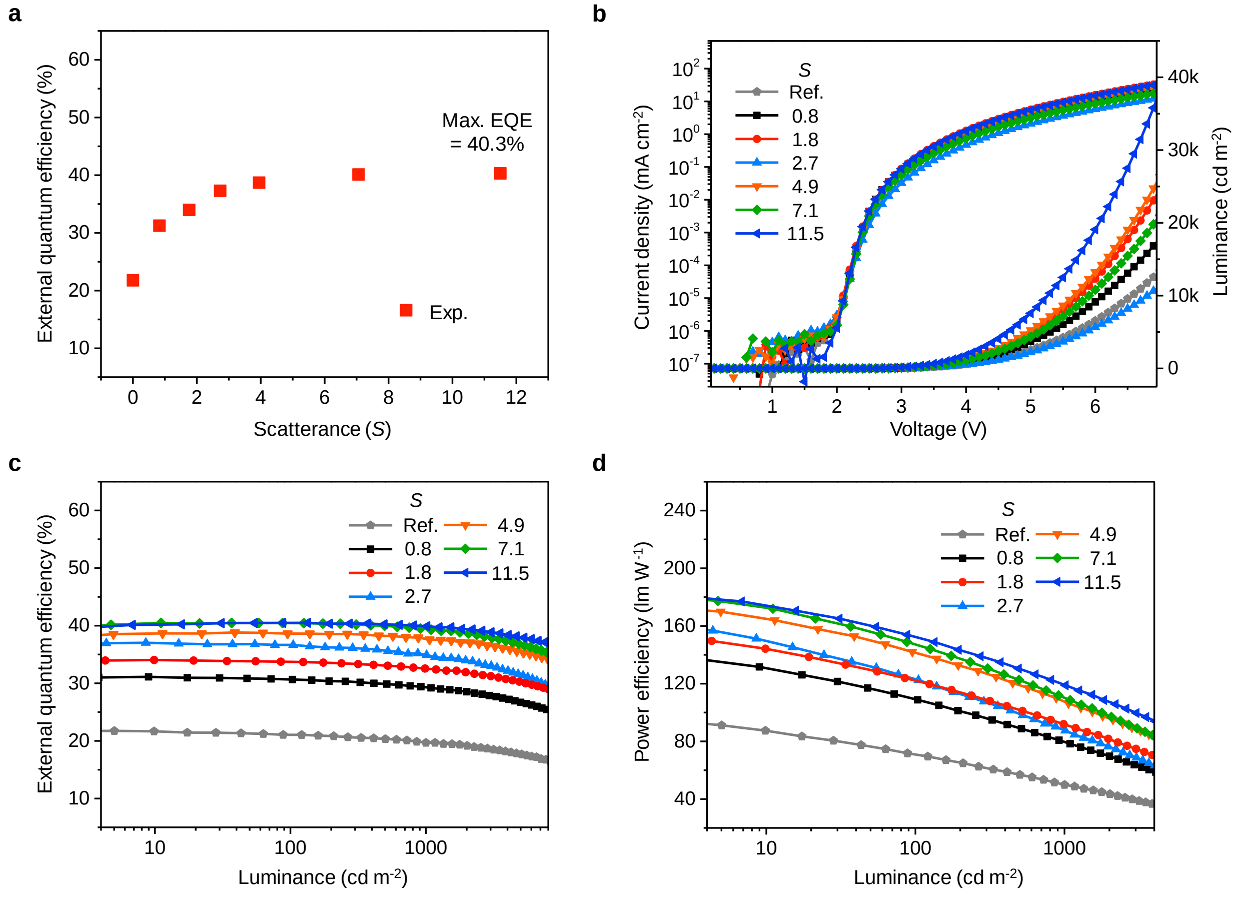

Supplementary Figure 9 | Characteristics of Ir(ppy)$_3$-based organic light-emitting diodes with SiO_2 scattering layers. (a) External quantum efficiency (EQE) as a function of scatterance (*S*) **(b)** Current density (*J*) - luminance (*L*) - voltage (*V*) characteristics. **(c-d) (c)** EQE and **(d)** Power efficiency versus *L*. Results shown here are for Type A devices having optimized structure: ITO (150 nm) / TAPC (80 nm) / TCTA (10 nm) / TCTA:B3PYMPM:Ir(ppy)$_3$ (8 wt.%, 30 nm) / B3PYMPM (65 nm) / LiF (1 nm) / Al (100 nm). Note that Ir(ppy)$_3$ has a random, isotropic dipole orientation (horizontal dipole orientation (Θ) = 0.67).

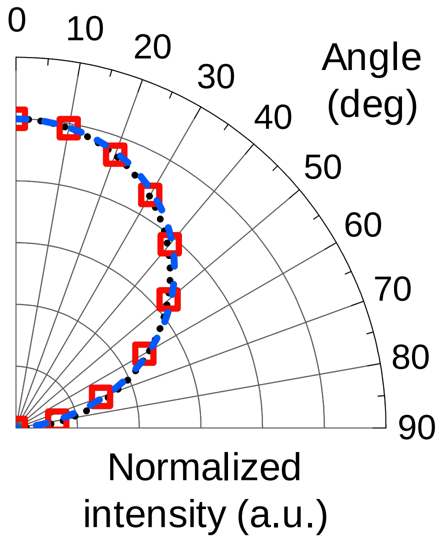

Supplementary Figure 10 | Normalized angular electroluminescence (EL) intensity. Normalized angular EL intensity (red hollow boxes) of OLEDs with a SiO_2 scattering layer, whose scatterance is 7.1, is compared with simulation result (blue dashed line). Black dotted line represents the Lambertian distribution.

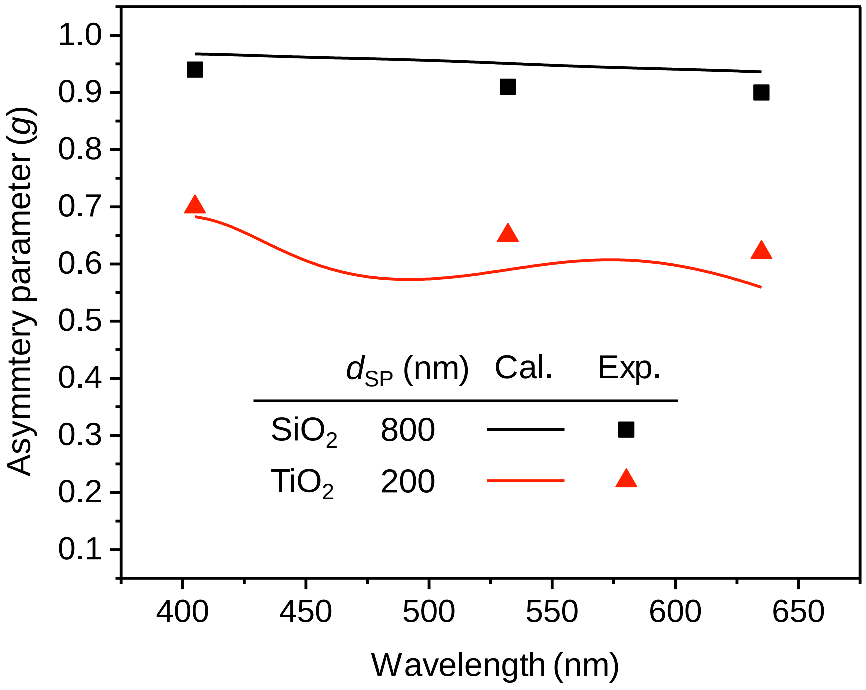

Supplementary Figure 11 | Asymmetry parameter vs. wavelength for the SiO_2 and TiO_2 particles embedded in NOA73 host.

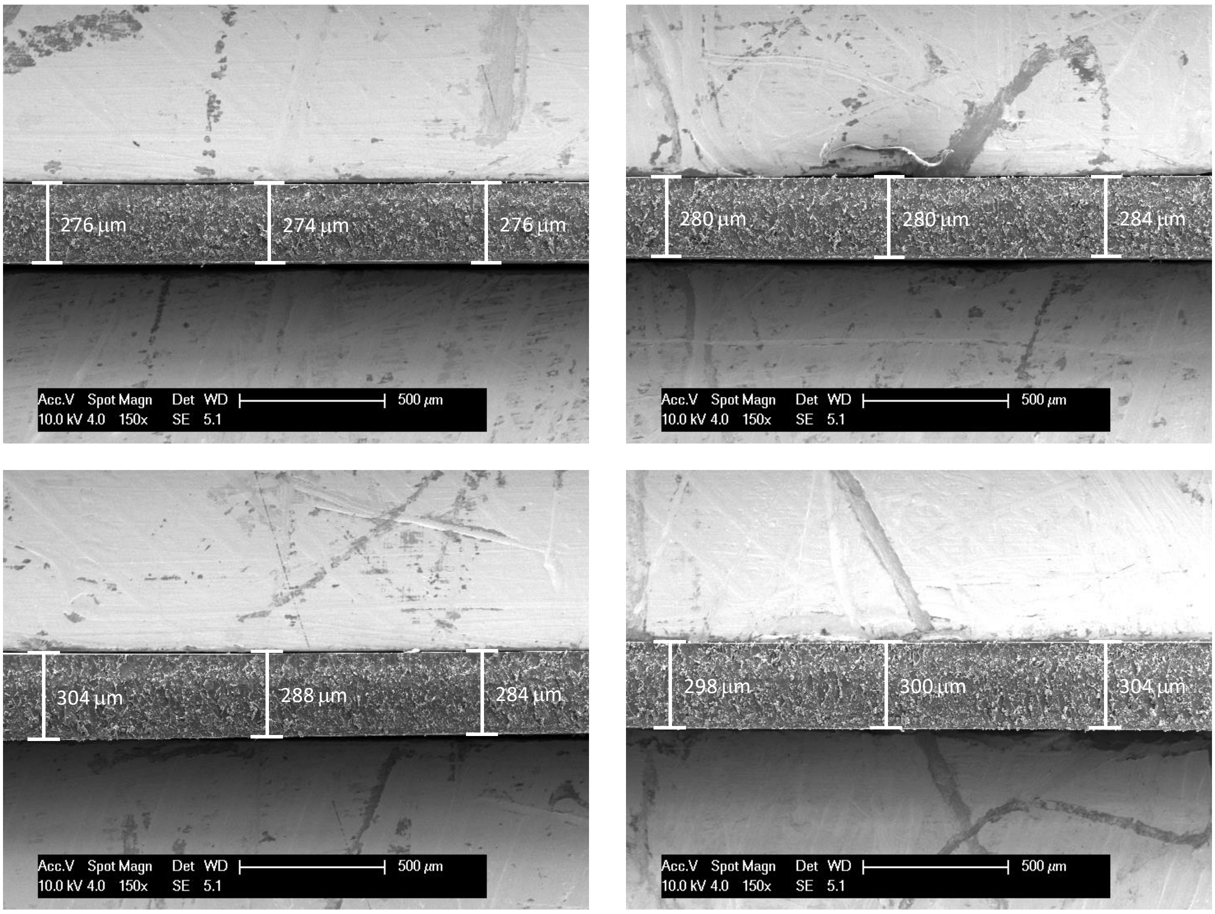

Supplementary Figure 12 | Scanning electron microscopy images of the cross-sections of scattering layers. The average thickness measured from 12 positions is 287 (± 11) μm.

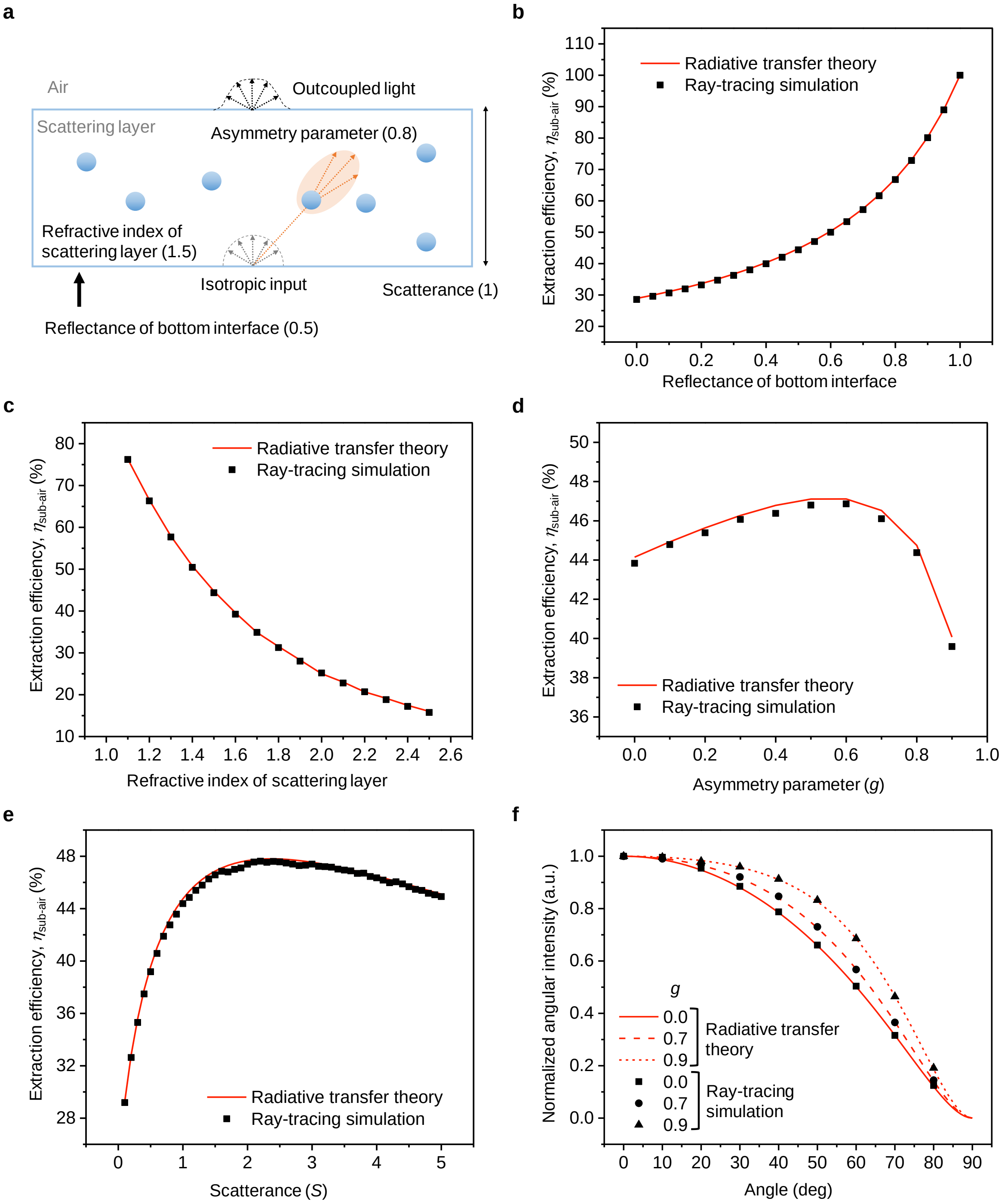

Supplementary Figure 13 | Comparison between simulation results from radiative transfer theory and ray-tracing simulation. **(a)** Simulation structure used for comparison. **(b-e)** Substrate-to-air extraction efficiency ($\eta_{\text{sub-air}}$) as a function of **(b)** Reflectance of bottom interface, **(c)** Refractive index of the scattering layer, **(d)** Scatterance (S), and **(e)** Asymmetry parameter (g) **(f)** Normalized angular intensity of the outcoupled light in case of g being 0.0, 0.7, and 0.9.

Supplementary References

1. Bohren, C. F. & Huffman, D. R. *Absorption and Scattering of Light by Small Particles* (John Wiley & Sons, 2008).

2. Kim, K-H., Ahn, E. S., Huh, J-S., Kim, Y-H. & Kim, J-J. Design of Heteroleptic Ir Complexes with Horizontal Emitting Dipoles for Highly Efficient Organic Light-Emitting Diodes with an External Quantum Efficiency of 38%. *Chem. Mater.* **28,** 7505-7510 (2016).
